# GPRC5A suppresses protein synthesis at the endoplasmic reticulum to prevent radiation-induced lung tumorigenesis

**DOI:** 10.1038/ncomms11795

**Published:** 2016-06-08

**Authors:** Jian Wang, Alton B. Farris, Kaiming Xu, Ping Wang, Xiangming Zhang, Duc M. Duong, Hong Yi, Hui-Kuo Shu, Shi-Yong Sun, Ya Wang

**Affiliations:** 1 Department of Radiation Oncology, Emory University School of Medicine and the Winship Cancer Institute, Emory University, Atlanta, Georgia 30322, USA; 2 Department of Pathology, Emory University School of Medicine and the Winship Cancer Institute, Emory University, Atlanta, Georgia 30322, USA; 3 Emory Integrated Proteomics Core and Biochemistry Department, Atlanta, Georgia 30322, USA; 4 Robert P. Apkaran Integrated Electron Microscope Core, Emory University, Atlanta, Georgia 30322, USA; 5 Department of Hemotology and Medical Oncology, Emory University School of Medicine and the Winship Cancer Institute, Atlanta, Georgia 30322, USA

## Abstract

GPRC5A functions as a lung tumour suppressor to prevent spontaneous and environmentally induced lung carcinogenesis; however, the underlying mechanism remains unclear. Here we reveal that GPRC5A at the endoplasmic reticulum (ER) membrane suppresses synthesis of the secreted or membrane-bound proteins including a number of oncogenes, the most important one being *Egfr*. The ER-located GPRC5A disturbs the assembly of the eIF4F-mediated translation initiation complex on the mRNA cap through directly binding to the eIF4F complex with its two middle extracellular loops. Particularly, suppression of EGFR by GPRC5A contributes significantly to preventing ionizing radiation (IR)-induced lung tumorigenesis. Thus, GPRC5A deletion enhances IR-promoted EGFR expression through an increased translation rate, thereby significantly increasing lung tumour incidence in *Gprc5a^−/−^* mice. Our findings indicate that under-expressed GPRC5A during lung tumorigenesis enhances any transcriptional stimulation through an active translational status, which can be used to control oncogene expression and potentially the resulting related disease.

## Abstract (web summary)

GPRC5A is a retinoic acid inducible gene that is preferentially expressed in lung tissue. Here the authors report that GPRC5A suppresses the translation of EGFR by interfering with the eIF4F complex assembly, thereby limiting lung tumorigenesis, particularly radiation-induced lung tumorigenesis.

## Graphical abstract

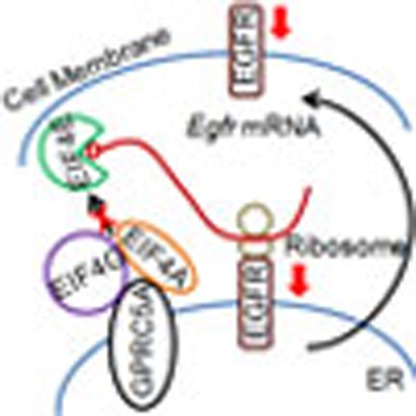

Lung tumours are the second most common tumours and the leading cause of cancer death in both men and women around the world, and adenocarcinoma is one of the most common lung tumours. On the basis of a published analysis of human data, G-protein-coupled receptor family C group 5 type A (GPRC5A) was significantly repressed in lung tumours, particularly in non-small-cell lung cancers (NSCLC)^1^. The GPRC5A gene locus is 12p13 and loss of heterozygosity of chromosome 12p was frequently found in NSCLC^2^,^3^. In addition, ∼10% of *Gprc5a* knockout mice spontaneously developed lung adenocarcinoma and lung cancer patients showed a significantly lower level of GPRC5A (ref. 1), indicating that GPRC5A is a lung tumour suppressor. However, the mechanism underlying how GPRC5A prevents lung tumorigenesis remains unclear. Investigation of GPRC5A-regulated gene expression will facilitate a better understanding of the role GPRC5A plays in preventing lung tumorigenesis.

Epidermal growth factor receptor (EGFR) is a key oncogene in lung adenocarcinoma^4^. EGFR is a transmembrane protein located in the cell surface membrane as well as in the nucleus^5^, which involves transcriptional regulation^6^,^7^, DNA replication and DNA repair^8^,^9^. Direct stimulation of EGFR by binding to a ligand, such as EGF, to the receptor's extracellular domain leads to dimerization and subsequent autophosphorylation of two receptor molecules, thereby creating phosphotyrosine docking sites that activate intracellular signalling cascades. It is well known, based on mine workers and atomic bomb survivors^10^,^11^, that ionizing radiation (IR) promotes lung tumorigenesis and abnormal EGFR is involved in radiation-stimulated lung cancers^12^; however, the whole picture needs to be elucidated. IR can stimulate the EGFR transcription, whereas only a moderate change in the protein level is induced by IR^13^, suggesting a strict control of EGFR expression aside from transcriptional control. Previous studies have shown a significant increase in EGFR expression when normal bronchial mucosa transforms epithelial hyperplasia and cancer^14^,^15^, suggesting that increasing EGFR expression may contribute to lung tumorigenesis in *Gprc5a^−/−^* mice.

Recently, it was reported that hypoxia/HIF2 activation could upregulate EGFR overexpression through increasing EGFR synthesis^16^, suggesting that the translation machinery plays an important role in EGFR regulation. In mammals, mRNA-independent translational regulation relies mainly on a direct modification of the translation initiation factors. The 43S pre-initiation complex binds to the messenger RNA (mRNA), which is thought to involve bridging interactions between eIF3 and the cap-binding eukaryotic initiation factor 4F (eIF4F) complex that is associated with the 5′-cap structure of the mRNA^17^. Alternated regulation of the eIF4F complex has been recently reported to play an essential role in carcinogenesis^18^,^19^. The eIF4F complex contains several proteins: eIF4E (it physically binds to the m7GpppN cap structure), eIF4A (a dead-box RNA helicase to unwind secondary structures in the 5′-UTR so that the 43S complex can bind and scan the mRNA^20^) and eIF4G that functions as a scaffold protein by interacting with eIF4E, eIF4A and eIF3 (ref. 21).

In this study, our data reveal a new regulation for EGFR by GPRC5A through translational suppression by directly binding to the eIF4F complex. Deletion of *Gprc5a* significantly enhances IR-stimulated EGFR expression due to loss of translational suppression, thereby causing an increase in IR-induced lung tumour incidence.

## Results

### GPRC5A downregulates EGFR expression post-transcriptionally

**Figure 1:**
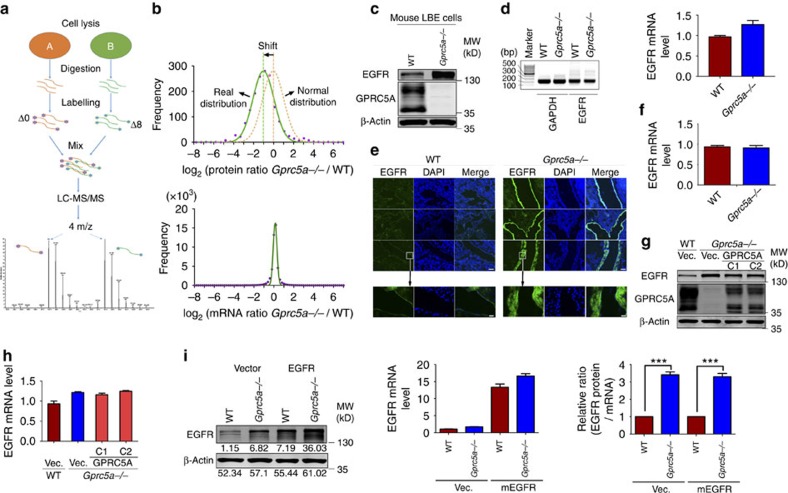
GPRC5A post-transcriptionally downregulates EGFR expression. (**a**) Flowchart of quantitative global proteomics using mTRAQ labelling. (**b**) Ratio distribution of *Gprc5a^−/−^* mouse LBE cells relative to wild-type LBE cells. The ratios shift the overall distribution (**—**) relative to representative normal distribution (---). (**c**) Western blot of EGFR, GPRC5A and β-Actin in wild-type and *Gprc5a^−/−^* LBE cells. (**d**) Semi-quantitative RT–PCR (left) and quantitative real-time PCR (right) of EGFR performed in triplicate using cDNAs obtained from wild-type and *Gprc5a^−/−^* LBE cells. (**e**) Images of immunofluorescence (IF) for EGFR in the lung frozen section of wild-type and *Gprc5a^−/−^* mice (top: Scale bar, 200 μm; bottom: Scale bar, 20 μm). (**f**) Real-time PCR of EGFR performed in biological triplicate using cDNAs obtained from wild-type and *Gprc5a^−/−^* lung tissue. (**g**) Western blot of EGFR, GPRC5A and β-Actin in wild-type and *Gprc5a^−/−^* LBE cells stably expressed with vector or GPRC5A (C1 and C2, respectively, represent Clone 1 and Clone 2). (**h**) Real-time PCR of EGFR performed in biological triplicate using cDNAs obtained from wild-type and *Gprc5a^−/−^* LBE cells stably expressed with vector or GPRC5A (C1 and C2, respectively, represent Clone 1 and Clone 2). (**i**) The relative ratio of EGFR protein and mRNA (right) calculated by western blot (left) and real-time PCR (middle) of EGFR in wild-type and *Gprc5a^−/−^* LBE cells stably expressed with vector or EGFR from three independent experiments (two-tailed Student's *t*-test, ***P<0.001). All western blots were shown are from a single experiment that is representative of at least three biological replicates. All the data are mean with s.e.m. from biological triplicate.

To understand how gene expression is regulated by GPRC5A at multiple levels in cells, we used a quantitative global proteomics approach by mTRAQ labelling (Fig. 1a) to identify the differentially expressed proteins between wild-type (*Gprc5a^+/+^)* and *Gprc5a^−/−^* mouse lung bronchial epithelial (LBE) cells. Interestingly, the quantitative analysis revealed a substantial perturbation of the cellular proteome, showing a marked distribution shift of quantified proteins relative to representative normal distribution (Fig. 1b, top panel and Supplementary Data 1), suggesting a structural change in the protein expression profile caused by GPRC5A deletion. Since there is no evidence of a decrease in the protein expression level in the center of the distribution curve (such as PCNA, XRCC5, SEC23a, XRCC1, actin and so on) by western blot analysis in *Gprc5a^−/−^* LBE cells compared with equal cell numbers of wild-type LBE cells, we excluded the possibility of a global downregulation of proteins by GPRC5A deletion. Thereby, we believe that GPRC5A deletion might cause a dramatic increase in the expression level of a specific group of proteins, resulting in a reduction on the proportion of other proteins in total proteins. Although the quantitative global proteomics data only includes a small portion of cell membrane proteins due to limitations in the approach, some cell membrane proteins in *Gprc5a^−/−^* LBE cells, such as EGFR, Tmem43, Cdh1, and Itgb4 and so on showed an extremely high expression, implying that GPRC5A might be involved in suppressing the cell membrane protein expression. Notably, the previously released mRNA microarray data of wild-type and *Gprc5a^−/−^* LBE cells (GSE21309) did not show a distribution curve shift (Fig. 1b, bottom panel) and the genes with significant protein level changes in the quantitative global proteomics data did not show corresponding mRNA level changes (Supplementary Data 1). In addition, the changes in the steady state of the mRNA levels versus corresponding proteins between wild-type and *Gprc5a^−/−^* LBE cells were plotted in Supplementary Fig. 1a. This analysis does not exhibit a correlation between changes in the steady state of the mRNA and protein levels in GPRC5A depleted versus control cells, suggesting that GPRC5A depletion affects the proteome in the absence of the major effect on the steady state of the mRNA levels. Altogether, these results suggest a post-transcriptional regulation of membrane proteins by GPRC5A.

EGFR is one of the most significantly increased proteins by the proteomic quantitative analysis in *Gprc5a^−/−^* LBE cells but without mRNA changes (Supplementary Data 1). The western blot of mouse LBE cells verified a much higher EGFR protein level in the *Gprc5a^−/−^* LBE cells (Fig. 1c), and mRNA levels measured by semi-quantitative PCR with reverse transcription (RT–PCR) or quantitative real-time PCR did not show a significant difference between wild-type and *Gprc5a^−/−^* LBE cells (Fig. 1d). In addition, the deletion of GPRC5A did not cause any changes in *Egfr* mRNA turnover rates (Supplementary Fig. 1b). Consistent with the data from LBE cells, a stronger EGFR staining was observed in the lung tissue of *Gprc5a^−/−^* mice compared with that of their wild-type counterparts (Fig. 1e), where the GPRC5A expression was examined by immunohistochemistry (IHC) and Laz staining^1^ (Supplementary Fig. 1c, d). Also, the *Egfr* mRNA level did not show any difference between wild-type and *Gprc5a^−/−^* mouse lung tissue (Fig. 1f). Next, complementary expression of GPRC5A in *Gprc5a^−/−^* mice LBE cells markedly decreased the EGFR expression (Fig. 1g), but did not affect the *Egfr* mRNA level (Fig. 1h). Taken together, these results suggest a post-transcriptional regulation of EGFR by GPRC5A. To further verify this, we compared the relative ratio of the EGFR protein level with the *Egfr* mRNA level in wild-type and *Gprc5a^−/−^* mouse LBE cells. The relative ratio of EGFR protein to its mRNA was around 4-fold higher in *Gprc5a^−/−^* than in wild-type LBE cells, and exogenous expression of EGFR or knockdown of *Egfr* with siRNA in wild-type and *Gprc5a^−/−^* LBE did not change the ratio (Fig. 1i; Supplementary Fig. 1e). Thus, these results provide strong evidence that GRPC5A downregulated EGFR expression in mouse LBE cells functions in a post-transcriptional manner.

The phosphorylated EGFR signal in *Gprc5a^−/−^* LBE cells was much greater than in wild-type LBE cells after treating with EGF (Supplementary Fig. 1f), indicating a larger response capability to the EGF signal in *Gprc5a^−/−^* LBE cells. These results also provide another explanation for constant activation of EGFR in the lungs of *Gprc5a^−/−^* mice^22^. Here we found that EGFR in wild-type and *Gprc5a^−/−^* LBE cells are located mainly at the cell surface membrane and partially in the nucleus (Supplementary Fig. 1g,h). These results indicate that the increased EGFR, a result of GPRC5A deletion, maintains its function and cellular distribution. Consistent with the mouse data, the EGFR protein level markedly decreased in GPRC5A-expressed H1299 cells compared with vector-expressed H1299 cells (human lung tumour cell line with a critically low GPRC5A expression), whereas the *Egfr* mRNA level did not change significantly (Supplementary Fig. 1i). The relative ratio of EGFR protein to mRNA in vector-expressed H1299 cells was >10-fold higher than in GPRC5A-expressed H1299 cells (Supplementary Fig. 1i). In conclusion, the above results indicate that GPRC5A disturbs the EGFR signalling pathway via post-transcriptional downregulation of EGFR expression in both mouse and human LBE cells.

### GPRC5A downregulates EGFR level via inhibiting translation

**Figure 2:**
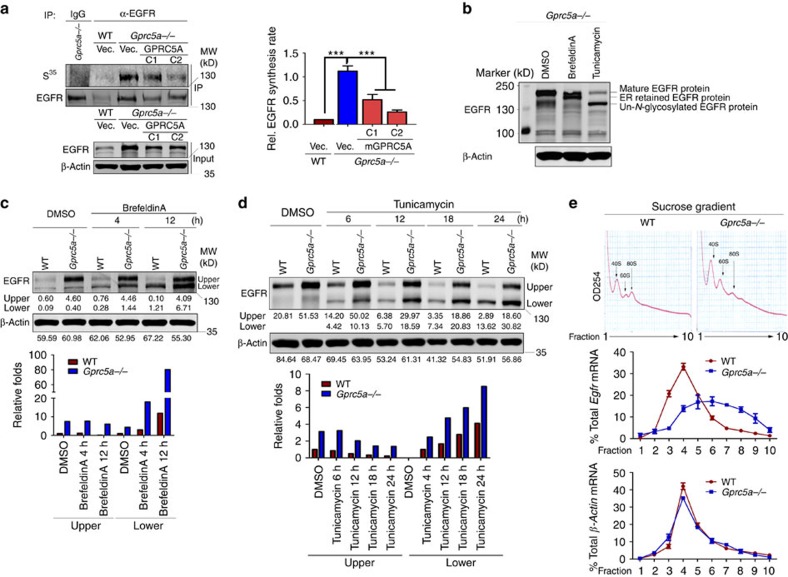
GPRC5A downregulates EGFR expression through suppressing translation. (**a**) EGFR translation rates of wild-type and *Gprc5a^−/−^* LBE cells stably expressed with vector or GPRC5A measured by [^35^S] methionine–cysteine incorporation (C1 and C2, respectively, represent Clone 1 and Clone 2). Left: image of phosphor and western blot; Right: bar graphic of the value of each band from triplicate independent experiments using ImageJ (two-tailed Student's *t*-test, ***P<0.001). (**b**) Western blot of EGFR and β-Actin in wild-type LBE cells after Brefeldin A (100 ng ml^−1^) or Tunicamycin (1 μg ml^−1^) treatment at indicated time points. (**c**) Western blot of EGFR and β-Actin in wild-type and *Gprc5a^−/−^* LBE cells after Brefeldin A treatment at indicated time point (Top), and graphic representation of the data obtained by western blot analysis (bottom). (**d**) Western blot of EGFR and β-Actin in wild-type and *Gprc5a^−/−^* LBE cells after Tunicamycin treatment at indicated time points (Top), and graphic representation of the data obtained by western blot analysis (bottom). (**e**) Polysomal distribution of EGFR mRNA in wild-type and *Gprc5a^−/−^* LBE cells. Top: A254 absorption of fractions. Bottom: *Egfr* and *β-Actin* mRNA polysomal distribution obtained by a real-time PCR analysis of biological triplicate. All western blots were shown are from a single experiment that is representative of at least three biological replicates. All the data are mean with s.e.m. from biological triplicate.

To investigate the underlying mechanism of post-transcriptional regulation of EGFR by GPRC5A, we initially examined the protein degradation rates of EGFR in wild-type and *Gprc5a^−/−^* LBE cells by treating the cells with a protein synthesis inhibitor, cycloheximide. There was no difference in the EGFR protein degradation rates between wild-type and *Gprc5a^−/−^* LBE cells (Supplementary Fig. 2a). In addition, none of the proteasome inhibitors (MG132 and Leupeptin), lysosome inhibitor (NH_4_Cl or Choroquine) or autophagy inhibitor (Bafilomycin A1) treatment could rescue the decrease in EGFR caused by GPRC5A (Supplementary Fig. 2b). Altogether, these results indicate that GPRC5A does not affect EGFR protein degradation, implying a translational regulation of EGFR by GPRC5A. The [^35^S] methionine–cysteine incorporated results showed a sharp increase in [^35^S]-labelled EGFR in *Gprc5a^−/−^* LBE cells as compared with that in wild-type LBE cells (Fig. 2a). And, this increased incorporation was rescued by the complementary expression of GPRC5A in *Gprc5a^−/−^* LBE cells (Fig. 2a), which suggests that GPRC5A is involved in the translational regulation of EGFR. In addition, we developed a novel approach to examine the newly synthesized EGFR protein by generating EGFR with a lower molecular weight (newly synthesized EGFR without modification) in the cells treated with Brefeldin A (inhibiting protein transport from the endoplasmic reticulum (ER) to the Golgi apparatus, causing the newly synthesized EGFR to lack further modification (especially glycosylation) in Golgi apparatus) or Tunicamycin (blocking *N*-linked glycosylation, causing the newly synthesized EGFR to lack *N*-linked glycosylation; Fig. 2b). These results further confirm that EGFR has a higher translation rate in *Gprc5a^−/−^* LBE cells than in wild-type LBE cells treated with Brefeldin A (Fig. 2c) or Tunicamycin (Fig. 2d). More importantly, deletion of GPRC5A resulted in a marked shift of the *Egfr* mRNA from monosomes to actively translating polysomes, indicating an active translation status of *Egfr* in *Gprc5a^−/−^* LBE cells (Fig. 2e). Altogether, these findings indicate a translational regulation of EGFR by GPRC5A.

### GPRC5A at ER membrane suppresses EGFR synthesis

To study how GPRC5A suppresses EGFR synthesis, we examined the luciferase activities between wild-type and *Gprc5a^−/−^* LBE cells after transfecting the reporter vector encoding the 3′-untranslated regions (UTRs) of *Egfr* at the downstream of the luciferase-coding sequence since previous studies indicated that EGFR translation was regulated on hypoxia through 3′-UTR-mediated HIF-2a–RBM4–eIF4E2 formation^16^,^23^, as well as by miR-7 (ref. 24). Again, there was no difference in the luciferase activities between wild-type and *Gprc5a^−/−^* LBE cells after transfecting the reporter vector encoding the 3′-UTR of *Egfr* at the downstream of the luciferase-coding sequence (Supplementary Fig. 3a). Therefore, these results exclude that GPRC5A regulates *Egfr* translation via 3′-UTR and suggests an mRNA-independent translational regulation.

**Figure 3:**
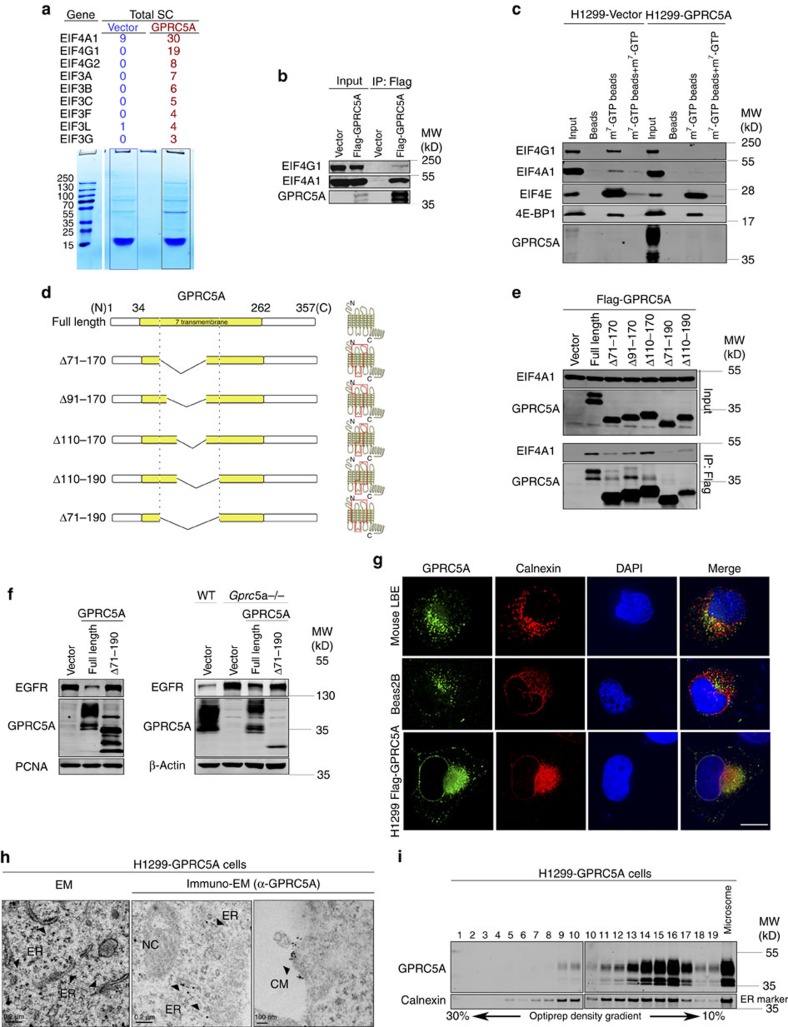
GPRC5A at ER suppresses *Egfr* translation through interacting with eIF4F. (**a**) Mass-spectrometry coupled with one-dimensional polyacrylamide gel separated by captured Flag-tagged human GPRC5A proteins and their interacting proteins from H1299 cells stably expressed Flag-GPRC5A. Bottom: image of coomassie blue R-250 staining; Top: list of eIFs candidates. (**b**) Co-immunoprecipitation (co-IP) of Flag-GPRC5A in H1299 cells. (**c**) Captured assays using agarose, cap-agarose (m^7^GTP immobilized to agarose by covalent linkage) or cap-agarose with free m^7^GTP addition in H1299 cells stably expressed vector or GPRC5A. (**d**) 2D graphic model of the main GPRC5A deletions located in seven transmembrane domains (left); and 3D model of the deletions (red box indicates the deletion localization) (right). (**e**) Co-immunoprecipitation (co-IP) of full-length Flag-GPRC5A or the indicated deletions in H1299 cells. (**f**) Left: western blot of EGFR, GPRC5A and PCNA in H1299 cells stably expressed vector, GPRC5A or GPRC5A(Δ71–190); Right: western blot of EGFR, GPRC5A and β-Actin in wild-type LBE cells expressed vector and *Gprc5a^−/−^* LBE cells expressed vector, GPRC5A or GPRC5A(Δ71–190). (**g**) Images of immunofluorescence (IF) for GRPC5A and Calnexin (ER marker) in wild-type LBE cells and Bease2B cells (Top 2), and Flag in H1299 cells expressed Flag-GPRC5A (bottom; Scale bar, 5 μm). (**h**) Immuno-transmission electron microscopy (Immuno-EM) of GPRC5A in H1299 cells expressed GPRC5A (NC, Nuclei; CM, Cell membrane; left: Scale bar=0.2 μm; middle: Scale bar, 0.2 μm; and right: Scale bar, 100 nm). (**i**) ER isolation by OptiPrep from H1299 cells expressed GPRC5A following western blot of GPRC5A and Calnexin in each fraction. All western blots were shown are from a single experiment that is representative of at least three biological replicates.

To look for the factors responsible for the GPRC5A-regulated EGFR translation, we performed mass-spectrometry coupled with one-dimensional polyacrylamide gel separating the captured Flag-tagged GPRC5A proteins and their interacting proteins from GPRC5A-expressed H1299 cells. The mixtures of proteins in stained bands were identified and analysed (Fig. 3a; Supplementary Data 2). eIF members were enriched in the list, including eIF4A, G (top two in the list) and eIF3s. Then, the interaction of GPRC5A with eIF4G1 and eIF4A1 was verified by co-immunoprecipitation (co-IP; Fig. 3b). RNA was not involved in the interaction because an RNase cocktail treatment was included in our standard IP procedure. In addition, a previous report pertaining to the census of human soluble protein complexes^25^ provides additional evidence that there might be a physical interaction between GPRC5A and eIF4A1.

To study whether GPRC5A can disturb the eIF4F complex from binding to the 5′-cap of the mRNA through direct interaction with the eIF4F complex, we isolated the cap-binding complexes from vector or GPRC5A-expressed H1299 lysates using cap-agarose (m^7^GTP immobilized to agarose by covalent linkage) and analysed the levels of eIF4G1, eIF4A1 and eIF4E in the cap-binding complexes. eIF4G1, eIF4A1 and eIF4E were detected in m^7^GTP-agarose precipitate and the addition of free m^7^GTP to the binding reactions prevented eIF4F precipitation in vector-expressed H1299 cells (Fig. 3c). 4E-BP1 is an important regulator for the eIF4F complex and its modification by mTORC1 significantly affects translation efficiency^26^,^27^. While expressing GPRC5A in H1299 cells, the binding of eIF4G1 and eIF4A1 to m^7^GTP-agarose was markedly attenuated; however, the binding of eIF4E and 4E-BP1 did not show any significant change (Fig. 3c). To examine whether GPRC5A-supressed *Egfr* translation was involved in mTORC1 factors/pathways, we compared the 4E-BP1 level and the status of some key factors in the mTORC1 signalling pathway between wild-type and *Gprc5a^−/−^* LBE cells. No significant differences in these factors were observed between wild-type and *Gprc5a^−/−^* LBE cells (Supplementary Fig. 3b), excluding the possibility of GPRC5A-supressed *Egfr* translation through these factors/pathways. Altogether, these results suggest that the translational suppression by GPRC5A is through disturbing the formation of eIF4F on the mRNA cap, that is, the binding of eIF4G1 and eIF4A1 to eIF4E. To identify the main region(s) of GPRC5A that are required for association with eIF4F, we designed a series of Flag-tagged GPRC5A complementary DNA (cDNA) constructs with different deletions and truncations (Fig. 3d), the main deletions were located in 7 transmembrane domains, which were subjected to co-IP. The results demonstrate that deletion of 71–190 amino acids from GPRC5A, resulted in the disruption of two intact middle extracellular loops, abrogating the interaction of GPRC5A to eIF4A1 (Fig. 3e and Supplementary Fig. 3c). We then purified the Flag-tagged full-length and Δ(71–190) GPRC5A to verify their *in vitro* interaction with eIF4F and the effects on disturbing the binding of eIF4F to the mRNA cap by using m^7^GTP-agarose purified cap-binding proteins from H1299 cells. The results indicate that purified Flag-tagged full-length GPRC5A protein bound and disassociated cap-binding proteins from m^7^GTP-agarose *in vitro* but the Δ(71–190) protein could not (Supplementary Fig. 3d,e), further supporting that the interaction with eIF4F is essential for GPRC5A to disturb the binding of eIF4F to the mRNA cap. The region of 71–190 amino acids is highly conserved from human to mouse (Supplementary Fig. 3f); suggesting that the region is functionally involved in translation regulation. We then generated the Δ(71–190) GPRC5A-expressed human H1299 and mouse *Gprc5a^−/−^* LBE cell lines to test their effects on EGFR expression by comparing the full-length GPRC5A-expressed cell lines. Expression of the Δ(71–190) GPRC5A in both mouse *Gprc5a^−/−^* LBE and human H1299 cells lost the suppression of EGFR expression as compared with the full-length GPRC5A (Fig. 3f). These results indicate that GPRC5A suppresses *Egfr* translation through its two middle extracellular loops within the 71–190 amino acids interacting with eIF4F to disturb the assembly of the eIF4F-mediated translation initiation complex on the mRNA cap.

GPRC5A is generally believed to be located and functions at the cell surface membrane. Thus, it is intriguing how a cell membrane protein directly regulates protein translation, which occurs in the cytoplasm or across the ER membrane. Subcellular fractionation analysis of human GPRC5A-expressed H1299 cells and mouse LBE cells supports that GPRC5A exists mainly in the membrane component, including the plasma membrane as well as all organelle membranes, except the nuclear membrane (Supplementary Fig. 3g). Surprisingly, the immunofluorescence staining demonstrated that GPRC5A is located mainly in the ER region of both mouse and human LBE cells, as well as of GPRC5A-expressed H1299 cells (Fig. 3g), and the ER isolation and immuno-transmission electron microscopy data further confirm the ER location of GPRC5A in both human and mouse cells (Fig. 3h,i; Supplementary Fig. 3h). Altogether, these results suggest that GPRC5A is located at the ER membrane to suppress EGFR translation. Meanwhile, a small portion of GPRC5A was detected in the cell surface membrane of GPRC5A-expressed H1299 cells (Fig. 3g, bottom panel and Fig. 3h, right panel), which might involve EGFR signalling at the cell surface membrane through direct EGFR binding as previously reported by Deng's group^22^. Our results reveal a novel mechanism whereby GPRC5A is involved in the EGFR signalling pathway, which provides further evidence that GPRC5A as a lung tumour suppressor is extensively involved in controlling oncogenic EGFR signalling, particularly in lung tumorigenesis.

### GPRC5A prevents IR-induced lung tumorigenesis

**Figure 4:**
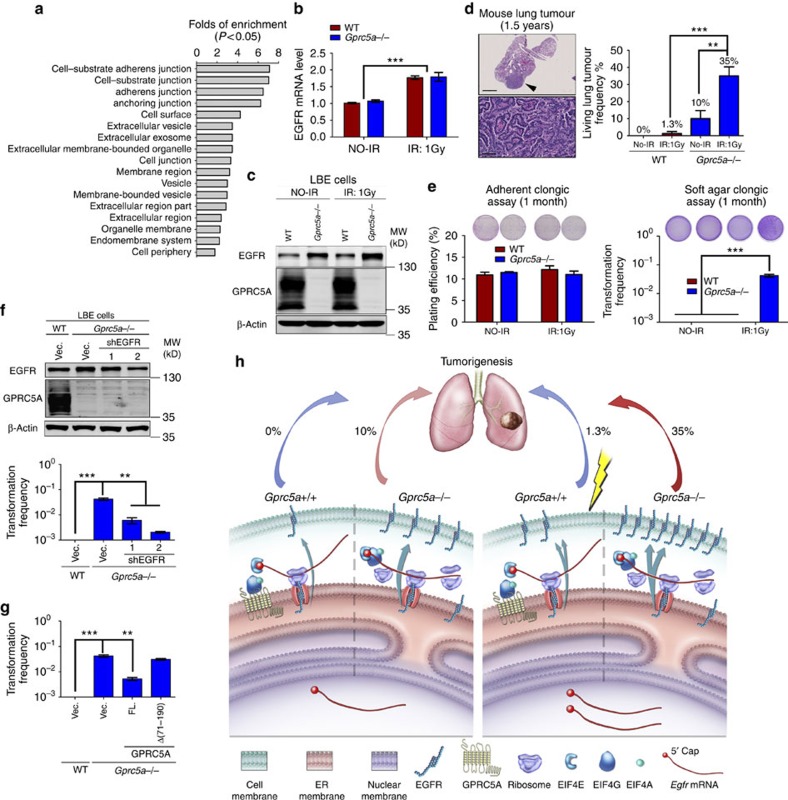
GPRC5A suppressing EGFR contributes to preventing IR-induced lung tumorigenesis. (**a**) Gene enrichment analysis by AmiGO 2 of the significant downregulated protein in GPRC5A-expressed H1299 cells compared to vector-expressed H1299 cells. (**b**) Real-time PCR of *Egfr* was performed in triplicate using cDNAs obtained from wild-type (WT) and *Gprc5a^−/−^* LBE cells at 1 month after sham irradiation (no-IR) or exposure to 1 Gy of X-ray (IR) (Student's *t*-test, ***P<0.001). (**c**) Western blot was derived from LBE cells at 1 month after 1 Gy IR. (**d**) Histological image (top: Scale bar, 3 mm; bottom: Scale bar, 50 μm) of mouse lung tumour with a local enlarged area (top) and statistical analysis of lung tumorigenesis in mice at 1.5 years after no-IR or exposure to 1 Gy IR (*n*=40 for each type of no-IR group; *n*=80 for each type of IR group) using the two-tailed *Z*-test (**P<0.01, ***P<0.001; Bottom). (**e**) Left: adherent clonogenic plating efficiency. Right: soft-agar clonogenic assay of LBE cells at 1 month after 1 Gy IR, statistical analysis between groups by two-tailed Student's *t*-test (***P<0.001). (**f**) Western blot was derived from LBE cells stably expressed with EGFR (shRNA1 or 2; top). Soft-agar clonogenic assay of LBE cells stably expressed with EGFR shRNA1 or 2 at 1 month after exposure to 1 Gy of X-ray and histogram of each samples from triplicate independent experiments with statistical analysis by two-tailed Student's *t*-test (***P<0.001; **P<0.01). (**g**) Soft-agar clonogenic assay of LBE cells stably expressed with full-length GPRC5A or Δ(71–190) GPRC5A at 1 month after exposure to 1 Gy of X-ray and histogram of each samples from triplicate independent experiments with statistical analysis by two-tailed Student's *t*-test (***P<0.001; **P<0.01; bottom). (**h**) Model depicting GPRC5A inhibits global translation at ER including EGFR, the key protein that GPRC5A relies on to prevent IR-induced lung tumorigenesis.

The above translational regulation implies that GRRC5A might be involved in regulating the repertoire translation that is destined to be secreted or membrane-bound proteins, which occurs across ER. To verify this, we compared the difference in protein levels between vector and GPRC5A-expressed H1299 cells using an mTRAQ quantitative proteomic approach and followed an enrichment analysis of the downregulated proteins in GPRC5A-expressed H1299 cells by AmiGO 2 (Supplementary Data 3). As expected, the genes encoding secreted or membrane-bound proteins were enriched (Fig. 4a; Supplementary Data 3). Next, antibody arrays (Human Growth Factor Antibody Arrays from Abcam and Proteome Profiler Human Receptor Array from R&D system) were introduced to examine these secreted or membrane-bound protein level changes between vector and GPRC5A-expressed H1299 cells. Strikingly, 8 of 40 proteins in the Human Growth Factor Antibody Arrays (EGFR is one of the top decreased proteins) and 20/105 proteins in the Proteome Profiler Humans Receptor Array (Supplementary Fig. 4a) obviously decreased by expressing GPRC5A in H1299 cells. Altogether, these results support that GPRC5A suppresses synthesis of the secreted or membrane-bound proteins at the ER membrane, which is the reason for a structural change in the protein expression profile caused by GPRC5A deletion (Fig. 1b, top panel). Among all identified genes translationally suppressed by GPRC5A, there are several oncogenes besides EGFR, such as HB-EGF, TGF-β, TGF-β2, VEGF and Integrin α/β. Deregulated translation control is a hallmark of human cancers and is critical for tumorigenesis downstream of multiple oncogenic signalling pathways^28^,^29^. However, considering the important role of EGFR in lung carcinogenesis, we believe that EGFR is the most important gene for *Gprc5a^−/−^* mice lung tumorigenesis, particularly after environmental stress stimulation.

To verify this, we first examined the effects of radiation on EGFR transcription in mouse LBE cells and mouse lung tissue. Both mouse LBE cells and lung tissue showed an increase in radiation-stimulated EGFR mRNA expression (Fig. 4b; Supplementary Fig. 4b). However, EGFR protein was only detected to significantly increase in *Gprc5a^−/−^* LBE cells or mouse lung after IR treatment (Fig. 4c; Supplementary Fig. 4c), supporting that the deletion of GPRC5A, indeed, markedly enhanced IR-stimulated EGFR expression. Depletion of GPRC5A significantly increased the proliferation rates of both mouse and human LBE cells, indicating a stimulation of EGFR on cell proliferation (Supplementary Fig. 4d,e). The lung tumorigenesis data showed that at 1.5 years after whole-body exposure to 1 Gy of IR (X-ray), lung adenocarcinoma genesis in *Gprc5a^−/−^* mice increased from 10 to 35% (Fig. 4d); whereas, wild-type mice only showed 1.3% lung adenocarcinoma^30^. Consistent with mouse lung tumorigenesis data, the soft-agar colony-forming efficiency reveals the significant effects of IR on the oncogenic cell transformation in *Gprc5a^−/−^* LBE cells at 1 month after exposure to 1 Gy, although there was no significant difference in adherent growth plating efficiency between wild-type and *Gprc5a^−/−^* LBE cells at the same time with the same treatment (Fig. 4e). Both *in vivo* and *in vitro* data suggest a synergistic association of the IR with deletion of GPRC5A on oncogenic cell transformation and mouse lung tumorigenesis. To verify EGFR is the key player for the IR-induced oncogenic transformation in *Gprc5a^−/−^* LBE cell, we generated an EGFR knockdown *Gprc5a^−/−^* LBE cell line using *Egfr* shRNA, which was subjected to a cell transformation assay at 1 month after exposure to IR. The results showed that the depletion of EGFR did significantly attenuate IR-induced oncogenic transformation of *Gprc5a^−/−^* LBE cells (Fig. 4f). In addition, introducing full-length GPRC5A but not the Δ(71–190) deletion into *Gprc5a^−/−^* LBE cells can obviously decrease IR-induced oncogenic transformation in *Gprc5a^−/−^* LBE cells (Fig. 4g). These results strongly support that GPRC5A prevents IR-induced lung tumorigenesis is mainly through the interaction of GPRC5A with the eIF4F complex to suppress *Egfr* translation.

GPRC5A expression not only inhibited IR-induced normal cells oncogenic transformation, but also suppressed some oncogenic characteristics in tumour cells. Expression of GPRC5A in H1299 cells significantly inhibited the EGFR downstream MAPK/ERK pathway although the AKT pathway and the level of PCNA (a proliferation marker) did not show any changes (Supplementary Fig. 4f). Consistent to the PCNA data, the proliferation rate in the GPRC5A-expressed human tumour cells did not significantly change although the EGFR level was markedly reduced (Supplementary Fig. 1i). These results indicate that EGFR-stimulated proliferation is significantly inhibited by GPRC5A in non-tumour LBE cells, and GPRC5A plays an important role in preventing normal cell oncogenic transformation. However, human lung tumour cells are heterogeneous and the proliferation status in different tumour cells may be affected by more complicated factors/pathways and are not solely affected by GPRC5A or EGFR. Therefore, the proliferation of different tumour cells may respond to GPRC5A expression differently. Next, to determine how GPRC5A affects tumour progress, we analysed the correlation between GPRC5A expression levels and lung cancer patient survival. The results showed that the patients with higher GPRC5A levels had a longer median overall in survival time than the patients with lower GPRC5A levels (Supplementary Fig. 4g). These results strongly support that GPRC5A as a tumour suppressor plays an important role in the earlier stage of lung tumour development.

## Discussion

In this study, we show that GPRC5A functions as a tumour suppressor to prevent IR-induced lung tumorigenesis, which involves GPRC5A at ER to suppress synthesis for a group of membrane-associated proteins and EGFR is the most important among them. Since translation is one of the most energy-consuming processes in cells and acts as a critical homeostatic mechanism, translational dysregulation leads to a number of pathological states including cancer^31^. There are a subset of mRNAs encoding proliferation, survival and tumour-promoting proteins including cyclins^32^, ornathinine decarboxylase (*ODC*)^33^, *VEGF*^34^, *MYC*^35^ and phosphoribosyl-pyrophosphate synthetase2 *(PRPS2)*^36^, which are referred to as eIF4E-sensitive mRNAs^21^. These mRNAs with long and highly structured 5′-UTR are more dependent on the unwinding activity of eIF4A (ref. 37), which are different from house-keeping mRNAs, such as GAPDH and β-actin, with short and low complex 5′-UTR, are less affected by changes in eIF4A (ref. 38) (for further details see ref. 21). In this study, we show that *Egfr* is an additional eIF4E-sensitive mRNA, where translation is regulated by GPRC5A. These results provide additional evidence indicating a close relationship between dysregulation of eIF4E-sensitive mRNAs and cancer development, and demonstrate the importance of maintaining normal regulation of the eIF4 complex in translation to prevent cancer development.

In this study, our results address the importance of controlling EGFR expression by GPRC5A in the early stages of tumorigenesis. Such a conclusion is also strongly supported by another group's report that human inflammatory lung tissue (a pathological change closely related to lung tumorigenesis) from their collected cases showed without exception lower GPRC5A expression associated with higher EGFR expression^22^. Using an IR-stimulated mouse lung tumorigenesis model, we explain that IR enhances the EGFR transcription, which causes a moderate EGFR protein increase in LBE cells, whereas, loss of translational suppression in *Gprc5a^−/−^* LBE cells markedly amplifies the increase of the EGFR protein level after IR exposure. The significant increase in the EGFR protein level contributes significantly to IR-induced lung tumorgenesis in *Gprc5a^−/−^* mice (Fig. 4h). It is known that GPRC5A expression could significantly increase after retinoid acid (one type of vitamin A) treatment^39^. Our findings in this study not only unveil a new general role of GPRC5A in regulating protein synthesis, which provides a basic platform for studying the regulation of the translation machinery, but also provide direct experimental evidence that the potent translational regulation by increasing GPRC5A expression (stimulated by retinoid acid) can be utilized to control future oncogenic gene expression and related disease.

## Methods

### Mice and cells irradiation

*Gprc5a^+/+^* and *Gprc5a^−/−^* mice (∼6 weeks old, female and male are 1:1) with C57BL/6 background were obtained from Dr Lotan's lab^1^ that were bred and maintained in a conventional animal facility at our University. All animal experiments were in accordance with the Emory Institutional Animal Care and Use Committee (IACUC) policy and approved by Emory IACUC. C57BL/6 mice were ordered from Jackson Laboratory. No randomization and blinding was used in animal experiments. Mice were exposed to 1-Gy whole-body irradiation of X-ray using an X-ray machine (X-RAD 320, N. Branford 320 kV, 10 mA, the filtration with 1.5-mm aluminium, 0.8 mm tin, 0.25 mm copper for mice and 2 mm aluminium for cells) in our laboratory. The mice were killed at 1.5 years following irradiation and the lung organs were removed for pathological slide preparation. Mouse wild-type (*Gprc5a^+/+^*) and *Gprc5a^−/−^* LBE cells (spontaneous immortalized, from Dr Lotan's Lab) were grown in Ham's F12 complete growth medium (1.5 g l^−1^ sodium bicarbonate, 2.7 g l^−1^ glucose, 2.0 mM L-glutamine, 0.1 mM nonessential amino acids, 0.005 mg ml^−1^ insulin, 10 mg ml^−1^ epidermal growth factor, 0.001 mg ml^−1^ transferrin, 500 ng ml^−1^ hydrocortisone and 4% fetal bovine serum (FBS)). 293FT (transformed human embryo kidney cells, purchased from Invitrogen) was grown in DMEM supplemented with 10% FBS; Beas2B (transformed human LBE cells, purchased from ATCC) was grown in DMEM/Nutrient Mixture F-12 Ham supplemented with 10% FBS; H1299 (human NSCLC cells, purchased from ATCC) was grown in RPMI 1640 medium supplemented with 10% FBS. All cell lines used in this study were checked for mycoplasma contamination.

### Cell transformation

Cell transformation was measured by using a soft-agar colony-forming assay. One per cent of low melting temperature agarose and 2 × Ham's F12 complete growth medium were mixed to obtain a 0.5% agarose concentration and then, 2 ml of 0.5% agarose–NL20 complete medium mixture was added to each well in six-well plates and the agar was solidified at 4 °C. Cells were collected and mixed with Ham's F12 complete growth medium containing 0.7% agar to a final agar concentration of 0.35%. The cells were cultured at 37 °C with 5% CO_2_ for 3 weeks. The culture was stained with 0.2% p-iodonitrotetrazolium violet (Sigma) or scanned for colony counting, and colonies larger than 100 μm in diameter were counted. All the data are biological triplicate with technical duplicates each assay.

### Materials for cell transfection

Actinomycin D (A1410), Tunicamycin (T7765), cycloheximide (01810), MG132 (C221), Leupeptin (L9783), NH_4_Cl (254134), Choroquine (C6628) and Bafilomycin A1 (B1793) were purchased from Sigma. Brefeldin A (9972) was purchased from Cell signaling. *Egfr* siRNA was purchased from Santa Cruz Biotechnology Inc. siGENOME *Gprc5a* siRNAs was purchased from Dharmacon. TRC Lentiviral EGFR shRNA was purchased from Dharmacon. The sequences were shown in Supplementary Data 4. The target DNA fragments for plasmid construction were amplified using PCR with human and mouse cDNA library as a template, which were inserted into the suitable clone sites of vectors. All primers for construction were list in Supplementary Data 4. Vector plasmids: pCDH-CMV-MCS-EF1-Puro from System Biosciences; p3XFLAG-CMV-10 from sigma; psiCHECK-2 from Promega. Vector plasmids: pCDH-CMV-MCS-EF1-Puro from System Biosciences; p3XFLAG-CMV™-10 from sigma; psiCHECK-2 from Promega. All primers for construction were list in Supplementary Data 4. Plasmid and siRNA transfections were performed using Lipofectamine 3,000 (Invitrogen), according to the manufacturer's protocol.

### Lentiviruse packaging and stable cell lines generating

Lentiviruse packaging (pCDH-CMV-MCS-EF1-Puro constructs and TRC Lentiviral EGFR shRNA) and stable cell lines generating were performed using Lenti-Pac HIV Expression Packaging Kit (GeneCopoeia, HPK-LvTR-20) according to manufacturer's instruction.

### RT–PCR and real-time PCR

Total RNA was extracted from cells using miRNeasy mini kits (Qiagen). cDNA was synthesized using 500 ng of total RNA from each using SuperScript VILO cDNA Synthesis Kit (Life Technologies), followed by triplicate qPCR reactions using TaqMan assays (EGFR assay ID: Mm00433023_m1, internal control: GPAPDH) with the TaqMan Fast PCR Universal master mix on an Applied Biosystems 7500 Fast real-time PCR system. For semi-quantitative PCR, EGFR mRNA levels were measured using the primer (forward: 5′- ATTGGCTCCCAGTACCTCCT -3′ and reverse: 5′- ATTCCAAAGCCATCCACTTG -3′); GAPDH (forward primer: μ5′- AACTTTGGCATTGTGGAAGG -3′; reverse primer: 5- CACATTGGGGGTAGGAACAC -3′). All the data are biological triplicate with technical duplicates each assay.

### Protein detection

Western blot was performed using standard techniques. The antibodies are listed in Supplementary Data 4. For IP, cells were collected, lysed in NP-40 lysis buffer (50 mM Tris-HCl (pH 8.0), 150 mM NaCl, and 1% NP-40) including HALT protease and a phosphatase inhibitor cocktail (Thermo, # 78440) and RNase cocktail (Ambion, AM2286), sonicated, and centrifuged at 4 °C (10,000*g* for 15 min). The supernatant was incubated with antibodies for 2 h at 4 °C. Fifteen microlitres of ANTI-FLAG M2 Affinity Gel (sigma, F2462) was then added, and the mixture was incubated for 2 h at 4 °C. After five washes with lysis buffer, the gel was resuspended in sample buffer, and the protein samples were then subjected to a western blot. For an immunofluorescence assay, cells were fixed with 4% (wt/vol) paraformaldehyde (in 1 × PBS) for 10 min at room temperature, followed by permeabilization in 0.5% Triton X-100 (in 1 × PBS) for 10 min. Cells were first incubated with bovine serum albumin (3%) (in 1 × PBS) at 37 °C for 30 min and then incubated with antibodies against EGFR (Abcam, ab52894) Flag (Sigma, F1804), GPRC5A (Santa Cruz, SC-373824) (at a dilution of 1:1,000, 1:3,000 and 1:1,000) at 37 °C for 1 h. After washing with 0.5% Tween 20 (in 1 × PBS) three times for 10 min at room temperature, Alexa Fluor 488 Goat anti-Mouse IgG (H+L) Secondary Antibody and Alexa Fluor 568 Goat anti-Rabbit IgG (H+L) Secondary Antibody (at a dilution of 1:1,000) were added at 37 °C for 30 min. Nuclei were stained with DAPI. Cells were examined with a DeltaVision Deconvolution Microscope.

### Quantitative global proteomics

*Tissue homogenization and mTraq Labelling*. Each cell pellet was lysed in 200 μl of Urea lysis buffer (8 M urea, 100 mM NaHPO_4_, pH 8.5), including 2 μl (100 × stock) HALT protease and phosphatase inhibitor cocktail (Thermo). Protein supernatants were sonicated (Sonic Dismembrator, Fisher Scientific) three times for 5 s with 15-s intervals of rest at 30% amplitude to disrupt nucleic acids and subsequently vortexed. Protein concentration was determined by the bicinchoninic acid method, and samples were frozen in aliquots at −80 °C. Cell lysates (100 μg) were diluted with 50 mM NH_4_HCO_3_ to a final concentration of <2 M urea and then treated with 1 mM dithiothreitol at 25 °C for 30 min, followed by 5 mM iodoacetimide at 25 °C for 30 min in the dark. Protein was digested with 1:100 (w/w) lysyl endopeptidase (Wako) at 25 °C for 2 h and further digested overnight with 1:50 (w/w) trypsin (Promega) at 25 °C. Resulting peptides were desalted with a Sep-Pak C18 column (Waters) and dried under vacuum. Dried peptides were brought in 20 μl of dissolution buffer and the rest of the labelling was carried out according to the manufacturer's protocol. Briefly, isopropanol was added to the mTraq Reagent delta0 and delta8 and combined with the respective sample. The pH was check and the labelling was allowed to carry on for 1 h at room temperature. The labelled samples were then combining and desalting was carried out with another Sep-Pak C18 column and dried under vacuum.

*LC–MS/MS analysis*. The peptides were resuspended in peptide loading buffer (0.1% formic acid, 0.03% trifluoroacetic acid and 1% acetonitrile) to a concentration of 1 μg μl^−1^. Peptide mixtures were separated on a self-packed C18 (1.9 μm Dr Maisch, Germany) fused silica column (25 cm × 75 μM internal diameter; New Objective, Woburn, MA) by a Dionex Ultimate 3,000 RSLC-nano and monitored on an Orbitrap Fusion mass spectrometer (ThermoFisher Scientific, San Jose, CA). Elution was performed over a 140-min gradient at a rate of 400 nl min^−1^ with buffer B ranging from 3 to 80% (buffer A: 0.1% formic acid in water, buffer B: 0.08% formic in acetonitrile). The mass spectrometer cycle was programmed to collect in top speed mode with a cycle time of 5 s and parallelizable time on. The MS scans (400–1,600 *m*/*z* range, 200,000 AGC, 50 ms maximum ion time, 60% S-lens RF Level) were collected at a resolution of 120,000 at *m*/*z* 200 in profile mode and the MS/MS spectra (0.7 *m*/*z* isolation width, 30% collision energy, 10,000 AGC target, 50 ms maximum ion time) were acquired by the ion trap. Dynamic exclusion was set to exclude previous sequenced precursor ions for 20 s within a 10 p.p.m. window. Precursor ions with +1, and +8 or higher charged states were excluded from sequencing.

*MS data analysis*. The Proteome Discoverer (PD) software suite version 2.0 (ThermoFisher Scientific) was used to search and quantitate the MS/MS spectra to a complete fully tryptic NCBI Refseq *homo* sapiens target and a decoy database (version 62 with a total of 68,742 target entries). Searching parameters included mass tolerance ±10 p.p.m. for the precursor and 0.6 Da for the fragment ion, fully tryptic restriction, dynamic modifications for oxidized Met (+15.9949 Da), delta0 Lysine (+140.09496 Da), delta8 Lysine (+148.10916 Da), dynamic peptide N-terminal modifications for delta0 and delta8 (+140.09596 and +148.10916, respectively), dynamic protein N-terminal for acetylation (+42.03670), static modification for carbamidomethyl cysteine (+57.02146), 4 maximal internal modification sites and a maximum of two missed cleavages. Only b and y ions were considered for scoring. The filtering was performed with the Percolator PD node to a target peptide spectral match FDR of 1 per cent. Precursor quantitation was performed with the peptide and protein quantitation node and final protein ratio was calculated using the summed area intensity of the top 3 peptides.

### Identification of protein–protein interactions

The immuneprecipitation (IP) samples were resolved on a 10% SDS gel, and stained with Coomassie blue G250. The entire gel lane was then excised into 3 bands followed by in-gel digestion with 12.5 ng μl^−1^ of Trypsin (Promega, Madison, WI) at 37 °C overnight according to the manufacturer's protocol. Peptides were extracted with a solution of 5% formic acid and 50% acetonitrile and speed vacuumed to dryness. An equal volume of each peptide sample resuspended in loading buffer (0.1% formic acid, 0.03% trifluoroacetic acid and 1% acetonitrile) and peptide eluents were separated on a 15 cm 1.9 μm C18 (Dr Maisch, Germany) self-packed column (New Objective, Woburn, MA) by a NanoAcquity UHPLC (Waters, Milford, FA) and monitored on an Q-Exactive Plus mass spectrometer (ThermoFisher Scientific, San Jose, CA). Elution was performed over a 90 min gradient at a rate of 300 nl min^−1^ with buffer B ranging from 5 to 80% (buffer A: 0.1% formic acid and 5% dimethyl sulfoxide in water, buffer B: 0.1% formic and 5% dimethyl sulfoxide in acetonitrile). The mass spectrometer cycle was programmed to collect one full MS scan followed by 10 data dependent MS/MS scans. The MS scans were collected at a resolution of 70,000 (300–1,800 *m*/*z* range, 1,000,000 AGC, 100 ms maximum ion time) and the MS/MS spectra were acquired at a resolution of 17,500 (2 *m*/*z* isolation width, 25% collision energy, 10,000 AGC target, 50 ms maximum ion time). Dynamic exclusion was set to exclude previous sequenced peaks for 30 s within a 10-p.p.m. window. The SageN Sorcerer SEQUEST 4.3 algorithm was used to search and match MS/MS spectra to a complete fully tryptic NCBI Refseq *Homo sapiens* target and decoy database (version 62 with a total of 68,742 target entries). Searching parameters included mass tolerance of precursor ions (±20 p.p.m.) fully tryptic restriction, dynamic modifications for oxidized Met (+15.9949 Da), 3 maximal modification sites and a maximum of two missed cleavages. Only b and y ions were considered for scoring (Xcorr) and Xcorr along with ΔCn were dynamically increased for groups of peptides organized by a combination of chymotrypticity (fully or partial) and a precursor ion charge state to remove false-positive hits along with decoys until achieving a false-discovery rate (FDR) of<1%. The FDR was estimated by the number of decoy matches (nd) and total number of assigned matches (nt). FDR=2 × nd/nt, assuming mismatches in the original database were the same as in the decoy database.

### Immuno-transmission electron microscopy

Monolayer cells were fixed with 4% paraformaldehyde and 0.05% glutaraldehyde in 100 mM phosphate buffer (pH 7.4) for 30 min, followed by 1% paraformaldehyde in phosphate buffer at 4 °C overnight. The samples were then treated in 0.1% sodium borohydride in phosphate buffer for 15 min, permeabilized with 0.1 μg ml^−1^ digitonin in PBS and blocked with PBS containing 5% bovine serum albumin, 0.1% cold-water fish skin gelatin and 5% normal goat serum for 30 min. The samples were incubated with primary antibody and then second antibody at 4 °C overnight. For the silver enhancement, sections were agitated in Aurion R-gent SE-EM at room temperature for 2 h and then rinsed in ECS solution again. After additional rinses in PBS, samples were post fixed with 0.5% osmium tetroxide, dehydrated and embedded in Epon resin following the standard procedures.

### Cap-affinity chromatography

H1299 cell lysates were prepared using NP-40 lysis buffer. Cap-binding reactions were performed at 4 °C with 1 mg protein and 15 ml m^7^GTP agarose or agarose (GE Healthcare) in NT2 buffer (50 mM Tris-HCl (pH 7.4), 150 mM NaCl, 1 mM MgCl2, and 0.05% NP40] for one hour. Binding reactions were performed in the presence or absence or 0.1 mM cap analogues GpppG. Sepharose beads were washed three times with 1 ml NT2 buffer and then resuspended in sample buffer for SDS–PAGE. Antibodies to eIF4A (Abcam), eIF4G (Abcam), 4E-BP1(Cell signaling) and eIF4E (Cell signaling) were used for western blotting.

### [^35^S] methionine–cysteine incorporation experiment

Cells were starved for 30 min in Met/Cys-free medium, pulsed for 10 min with 200 μCi of 35S labelled Met/Cys in a 2-ml starvation medium/60-mm dish, and chased for the times indicated in the figures with the medium supplemented with 5 mM cold Met/Cys. Cells were lysed in 1 ml NP-40 lysis buffer containing 10 μl protease inhibitor cocktail (Thermo). Labelled proteins were immunoprecipitated from cell extracts using an EGFR (Millipore, 06-847) antibody. Immunoprecipitations were performed by adding protein A beads (Roche Diagnostics) and an Anti-EGFR antibody to the lysates. The immunoprecipitates were extensively washed three times with IP buffer, resuspended in sample buffer, resolved by SDS–PAGE and electrotransferred to PVDF. Blots were exposed to a Phospho image screen and scanned. Relevant bands were quantitated by phosphor imaging on a Typhoon 9210 system using ImageQuant software from Molecular Dynamics (subsidiary of GE Healthcare, Piscataway, NJ).

### Polysomal distribution

Cells were incubated with cycloheximide (100 μg ml^−1^) for 15 min to arrest polyribosome migration. Cells were then lysed in 800 μl of lysis buffer containing 20 mM Tris (pH 7.0), 100 mM KCl, 5 mM MgCl2, 0.5% Triton-X 100 and 8 μl protease inhibitor cocktail (Thermo), put on ice for 20 min and then spun at 13,000*g* at 4C for 30 min to isolate cytoplasmic extracts. Cytoplasmic extracts loaded on 15–45% (wt/vol) sucrose gradient were centrifuged at 39,000 g in a SW41 rotor for 60 min at 4 °C. After centrifuged, gradients were fractionated from the top by displacement through a flow cell, and A254 was monitored with a recording spectrophotometer. Total RNA was extracted from each fraction by miRNeasy serum/plasma kit (Qiagen, 217184) and RNA level was analysed by real-time PCR.

### Luciferase reporter assay

Wild-type and *Gprc5a^−/−^* LBE cells were transfected with the psiCHECK-2 vector or psiCHECK-2 containing mouse EGFR 3′-UTRs in 48-well plates. The cells were collected 48 h after transfection, and the cells were then lysed with a Dual-Glo luciferase assay System (Promega, E2920) according to the manufacturer's protocol and measured on a Berthold Detection System SIRIUS Single Tube Luminometer. Firefly luciferase was used for normalization. Each experiment was performed at least three times.

### Endoplasmic reticulum isolation

The ER isolation was conducted using an Endoplasmic Reticulum Isolation Kit (Sigma, ER0100) according to the manufacturer's protocol. Briefly, cells were suspended in a volume of 1 × Hypotonic Extraction Buffer equivalent to three times the packed cell volume (PCV) and incubated for 20 min at 4 °C to allow the cells to swell. Centrifuge the cells at 600*g* for 5 min and remove the supernatant by aspiration. Measure the ‘new' PCV. Add a volume of 1 × Isotonic Extraction Buffer equivalent to two times the ‘new' PCV and transfer to 7 ml Dounce homogenizer. Break the cells with 10 strokes of the Dounce homogenizer and centrifuge the homogenate at 1,000*g* for 10 min at 4 °C. Transfer the supernatant to another centrifuge tube. Centrifuge at 12,000*g* for 15 min at 4 °C. Transfer the supernatant to an ultracentrifuge tube for 60 min at 100,000*g* in an ultracentrifuge at 4 °C. The pellet is the crud microsomal fraction. Resuspend the pellet within 1 × Isotonic Extraction Buffer with appropriate homogenizer. The crude microsomal fraction is adjusted to 20% (w/v) Optiprep and is layered between 30 and 15% Optiprep layers. Following ultracentrifugation using a fixed angle rotor, fractions are separated from the top to the bottom of the gradient.

### Immunohistochemical analysis

IHC analysis using standard heat-induced epitope retrieval in citrate buffer (pH 6) was performed. Slides were loaded on the DAKO Autostainer (Dako, Carpenteria, CA), exposed to 3% hydrogen peroxide for 5 min, primary monoclonal antibody to GPRC5A (Santa Cruz, sc-98885) and EGFR (Abcam, ab52894) for 30 min, labelled with polymer horseradish peroxidase for 30 min, diaminobenzidine as a chromogen for 5 min, and haematoxylin as a counterstain for 5 min. Incubations were performed at room temperature; between the incubations, the sections were washed with Tris-buffered saline. Coverslipping was done using the Tissue-Tek SCA coverslipper (Sakura Finetek USA, Torrance, CA). A known EGFR positive control and negative control (Dako) were included in each run. IHC score equates the intensity of IHC staining multiplying percentage of positive cells.

### Subcellular fractionation

Subcellular fractionation was performed using Qproteome Cell Compartment kit (Qiagen, 37502) according to manufacturer's instruction.

### LacZ tissue staining

LacZ Tissue Staining kit (InvivoGen, rep-lz-t) was used to determine β-Galactosidase activity in intact tissues according to manufacturer's instruction.

### Antibody arrays

Human Growth Factor antibody array from Abcam (ab 134002) and Proteome Profiler Human sReceptor Array from R&D system (ARY012) were performed according to manufacturer's instructions.

### Statistical analysis

The Student's *t*-test was used for the comparison of two samples. *P* values <0.05 were considered significant. *Z*-test was used for test mean of a normally distributed population with known variance. Log-rank test was used for the comparison of two survival distributions samples. Error bars indicate s.e.m. The number of biological and experimental replicas ⩾3, otherwise mentioned in figure legends.

### Data availability

The authors declare that all the data supporting the findings of this study are available within the article and its Supplementary Information files.

## Additional information

**How to cite this article:** Wang, J. *et al*. GPRC5A suppresses protein synthesis at the endoplasmic reticulum to prevent radiation-induced lung tumorigenesis. *Nat. Commun.* 7:11795 doi: 10.1038/ncomms11795 (2016).

## Supplementary Material

Supplementary Information Supplementary Figures 1-5.

Supplementary Data 1 Raw data of quantitative global proteomics from mouse LBE cells.

Supplementary Data 2 Raw data of identification of protein-protein interactions by mass pectrometry.

Supplementary Data 3 Raw data of quantitative global proteomics from human H1299 cells.

Supplementary Data 4 includes the information of primers, antibodies (including source and dilution information) and siRNA/shRNA sequence.
