# Supplementary material for: GPRC5A suppresses protein synthesis at the endoplasmic reticulum to prevent radiation-induced lung tumorigenesis
Source: Nat Commun. 2016 Jun 8;7:11795. doi: 10.1038/ncomms11795 (PMC4899846; doi:10.1038/ncomms11795)
Supplement: Supplementary Information — Supplementary Figures 1-5. [file ncomms11795-s1.pdf]

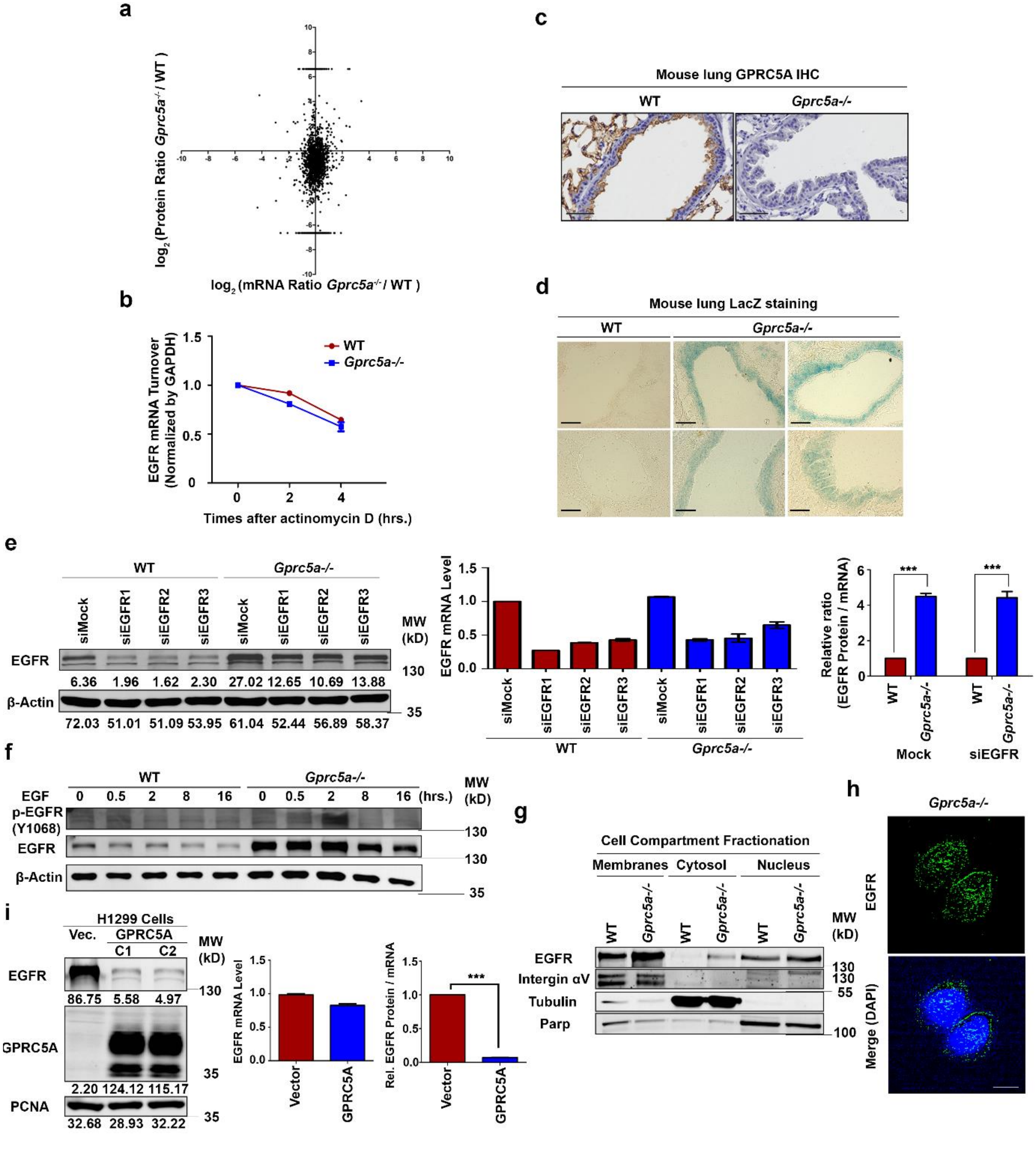


**Supplementary Fig. 1. GPRC5A post-transcriptionally down-regulates EGFR expression.** (**a**) Plot of the changes in steady state mRNA levels versus changes in corresponding proteins between wild type and *Gprc5a-/-* LBE cells (**b**) EGFR mRNA turnover rate normalized by GAPDH in wild type and *Gprc5a-/-* LBE cells: real time PCR of EGFR performed in biological triplicate using cDNAs obtained at indicated time points after Actinomycin D (10 µg/ml) treatment. (**c**) Images of IHC staining for GPRC5A in the lung of wild type and *Gprc5a-/-* mice (Scale Bar = 50 µm). (**d**) Detection of expression of the reporter gene LacZ driven by the endogenous Gprc5a promoter in

*Gprc5a-/-* mice using X-gal stain (Scale Bar = 50 μm). (**e**) The relative ratio of EGFR Protein / mRNA (Right) calculated by western blot (Left) and real time PCR (middle) of EGFR in wild type and *Gprc5a-/-* LBE cells transfected by mock or EGFR siRNA (triplicate) from 3 times independent experiments (Two-tailed Student's t-test, *** *P* <0.001). (**f**) Western blot of p-EGFR (Y1068), EGFR and β-Actin in wild type and *Gprc5a-/-* LBE cells after EGF treatment at indicated time point. (**g**) Western blot of EGFR in fractionated wild type and *Gprc5a-/-* LBE cells, proteins specific to each fraction were detected using integrin αV (membranes), Tubulin (Cytosol) and Parp (Nucleus). (**h**) Images of IF for EGFR in *Gprc5a-/-* LBE cells (Scale Bar = 5 μm). (**i**) Left: Western blot of EGFR, GPRC5A and PCNA in H1299 cells stably expressing vector or GPRC5A (C1 and C2 respectively represent Clone 1 and Clone 2). Middle: Real time PCR of EGFR in H1299 cells stably expressing vector or GPRC5A from 3 times independent experiments (Clone 2). Right: The relative ratio of EGFR Protein / mRNA measured by western blot and Real time PCR of EGFR in wild type and *Gprc5a-/-* LBE cells stably expressing vector or EGFR (Clone 2) from 3 times independent experiments (Student's t-test, *** *P* <0.001). All western blots shown are from a single experiment that is representative of at least 3 biological replicates. All data are mean with s.e.m from biological triplicate.

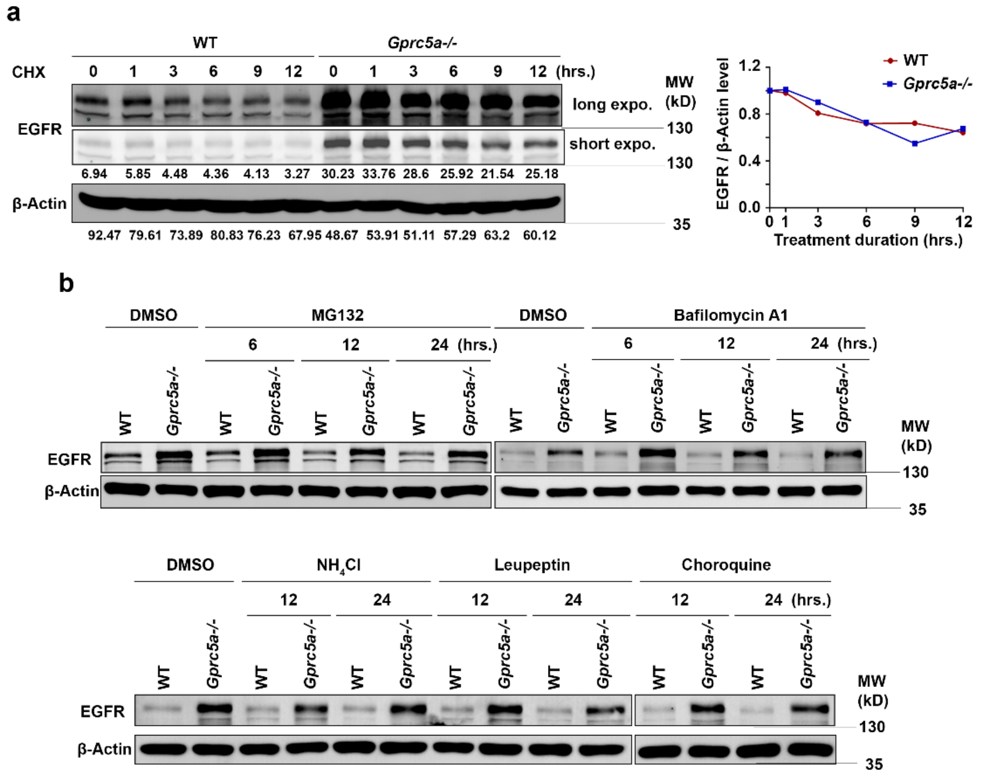


**Supplementary Fig. 2. GPRC5A down-regulates EGFR expression through suppressing translation.** (**a**) EGFR protein degradation rate normalized by β-Actin in wild type and *Gprc5a-/-* LBE cells (Left): Western blot of EGFR and β-Actin in wild type and *Gprc5a-/-* LBE cells after cycloheximide (CHX) (100 μg/ml) treatment at indicated time points (Right). (**b**) Western blot of EGFR and β-Actin in wild type and *Gprc5a-/-* LBE cells after MG132 (20 ng/ml), Leupeptin (50 μM), NH4Cl (25 mM), Choroquine (20 μM), or Bafilomycin A1 (50 nM) treatment at indicated time points. All western blots were shown are from a single experiment that is representative of at least 3 biological replicates.

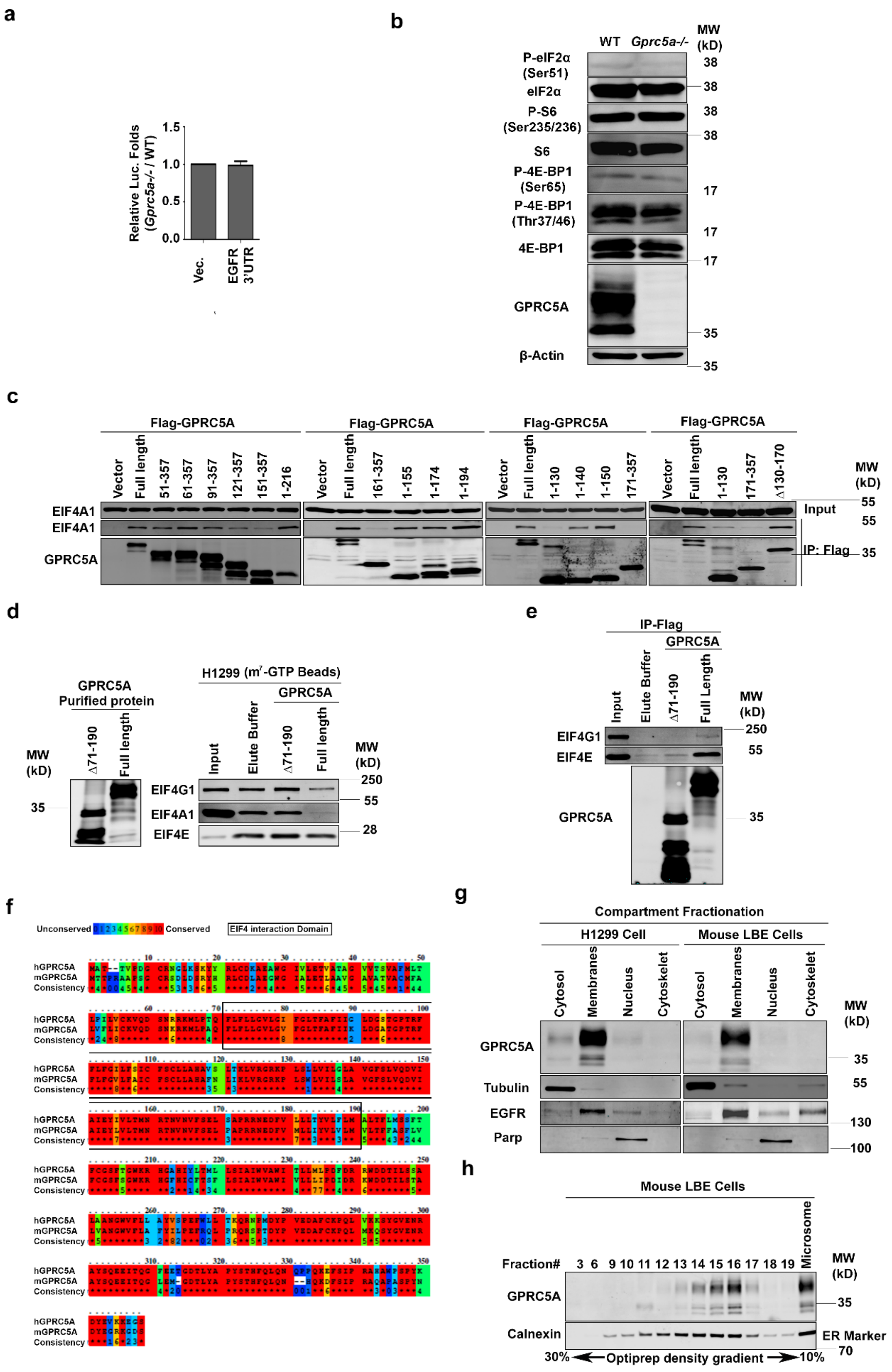


**Supplementary Fig. 3. GPRC5A at ER membrane suppresses EGFR translation**. (a) Luciferase assay of wild type and *Gprc5a-/-* LBE cells expressing psiCHECK$^{TM}$-2 (a luciferase

reporter vector) or psiCHECK$^{TM}$-2-EGFR3'UTR (biological triplicate). (**b**) Western blot of P-eIF2α (Ser51), eIF2α, P-S6 (Ser235/236), S6, P-4E-BP1 (Ser65), P-4E-BP1 (Thr37/46), 4E-BP1, GPRC5A and β-Actin in wild type and *Gprc5a-/-* LBE cells. (**c**) Co-immunoprecipitation (co-IP) of full length Flag-GPRC5A or the indicated truncations or deletions in H1299 cells. (**d**) GPRC5A disturbs the binding of eIF4F to cap-agarose *in vitro*: Add purified Flag-tagged full length GPRC5A or Δ(71-190) GPRC5A (protein elution buffer as control) to m$^7$GTP-agarose with purified cap-binding proteins from H1299 cells, mix, and wash, following with western blot of eIF4A, eIF4G and eIF4E in the precipitated agarose fraction. (**e**) GPRC5A binds to eIF4F *in vitro*: Add purified Flag-tagged full length GPRC5A or Δ(71-190) GPRC5A (protein eluted buffer as control) to m$^7$GTP-agarose with purified cap-binding proteins from H1299 cells and mix. Then, supernatant was subjected to co-IP of Flag, following with western blot of eIF4G and eIF4E in the precipitated fraction. (**f**) Alignment of human and mouse GPRC5A. The 71-190 amino acids of GPRC5A, which is the eIF4F interaction region, is shown in the black box. (**g**) Western blot of GPRC5A in fractionated H1299 cells and wild type LBE cells, proteins specific to each fraction were detected using EGFR (membranes), Tubulin (Cytosol) and Parp (Nucleus). (**h**) ER Isolation by OptiPrep from mouse wild type LBE cells following western blot of GPRC5A and Calnexin in each fraction. All western blots shown are from a single experiment that is representative of at least 3 biological replicates. All data are mean with s.e.m from biological triplicate.

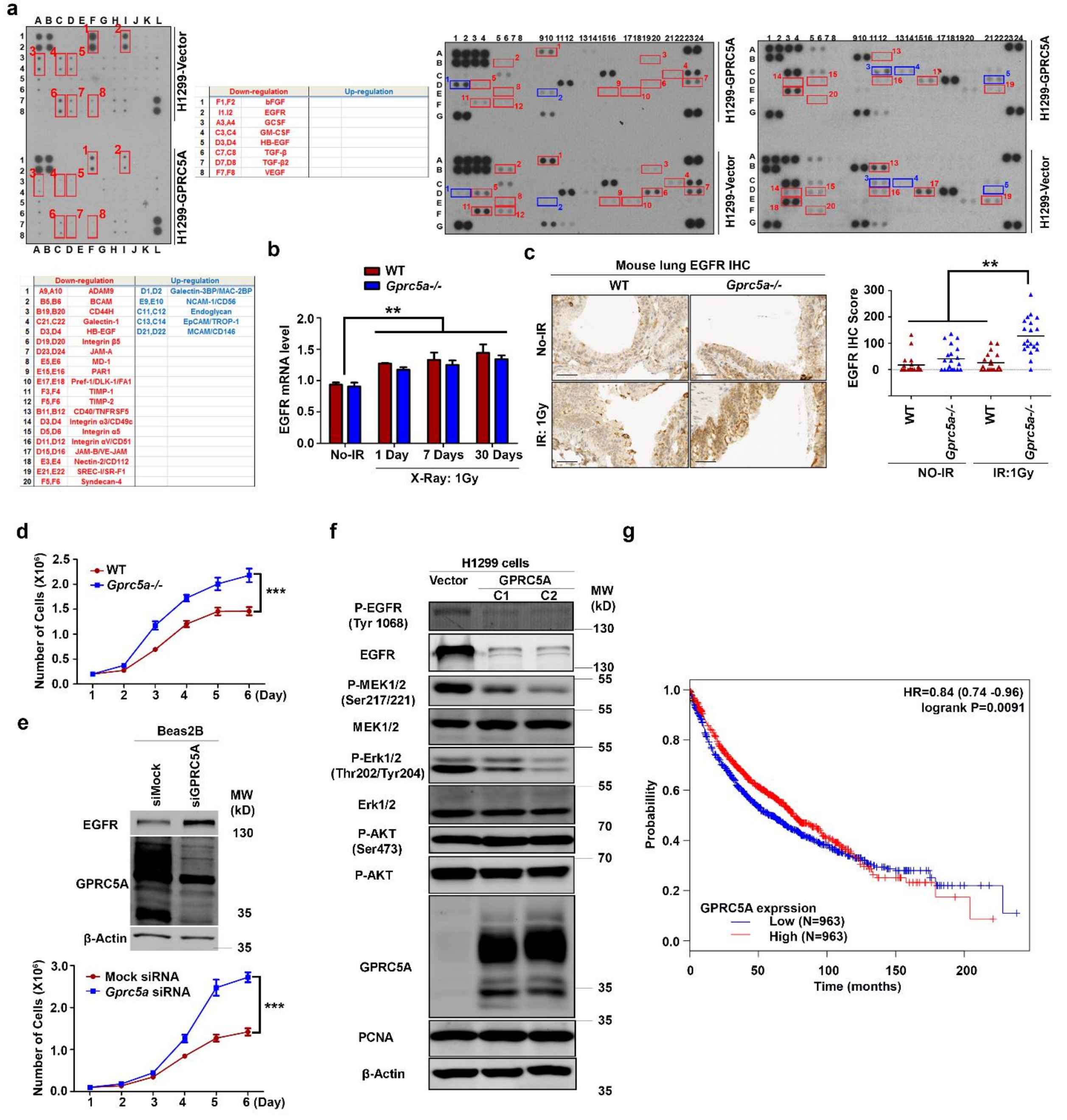


**Supplementary Fig. 4. GPRC5A suppressing EGFR contributes significantly to preventing IR-induced lung tumorigenesis.** (**a**) Antibody arrays: Human Growth Factor antibody arrays from Abcam (Left) and Proteome Profiler Human sReceptor Array from R&D system (Right) of a series well-known secreted growth factor and membrane-bound receptors in H1299 cells stably expressing vector or GPRC5A. The list of up- or down-regulated proteins in H1299 cells stably expressing GPRC5A comparing to H1299 cells stably expressing vector is also shown. (**b**) Real time PCR of EGFR was performed using cDNAs obtained from wild type and *Gprc5a-/-* mice (5 mice each group) lung tissue at indicated time point after sham irradiation or exposure to 1Gy of X-ray (Two-tailed Student's t-test, ** *P* <0.01). (**c**) Images of IHC staining for EGFR in the lung of wild type and *Gprc5a-/-* mice (20 mice each groups) at 1 month after sham irradiation or exposure to 1Gy of X-ray (Scale Bar = 50 µm) (left) and Whisker box plot (Two-tailed Student's

t-test, ** $P < 0.01$) (right). (d) Cell growth curve of wild type and *Gprc5a-/-* LBE cells (Biological triplicate). (e) Top: Western blot of EGFR, GPRC5A and β-Actin in mock siRNA and GPRC5A siRNA transfected Beas2B cells; Bottom: Cell growth curve of mock siRNA and GPRC5A siRNA transfected Beas2B cells (Biological triplicate). (f) Western blot of P-EGFR (Tyr 1068), EGFR, P-MEK1/2 (Ser217/221), MEK1/2, P-Erk1/2 (Ser202/Tyr204), Erk1/2, P-AKT (Ser473), AKT, GPRC5A and β-Actin in cells stably expressing vector or GPRC5A (C1 and C2 respectively represent Clone 1 and Clone 2). (g) Kaplan-Meier survival curve for overall survival of Lung cancer patients with low and high expression of GPRC5A with Log rank test. (Using online Kaplan-Meier Plotter tools: http://kmplot.com/analysis/). All western blots were shown are from a single experiment that is representative of at least 3 biological replicates. All data are mean with s.e.m from biological triplicate.

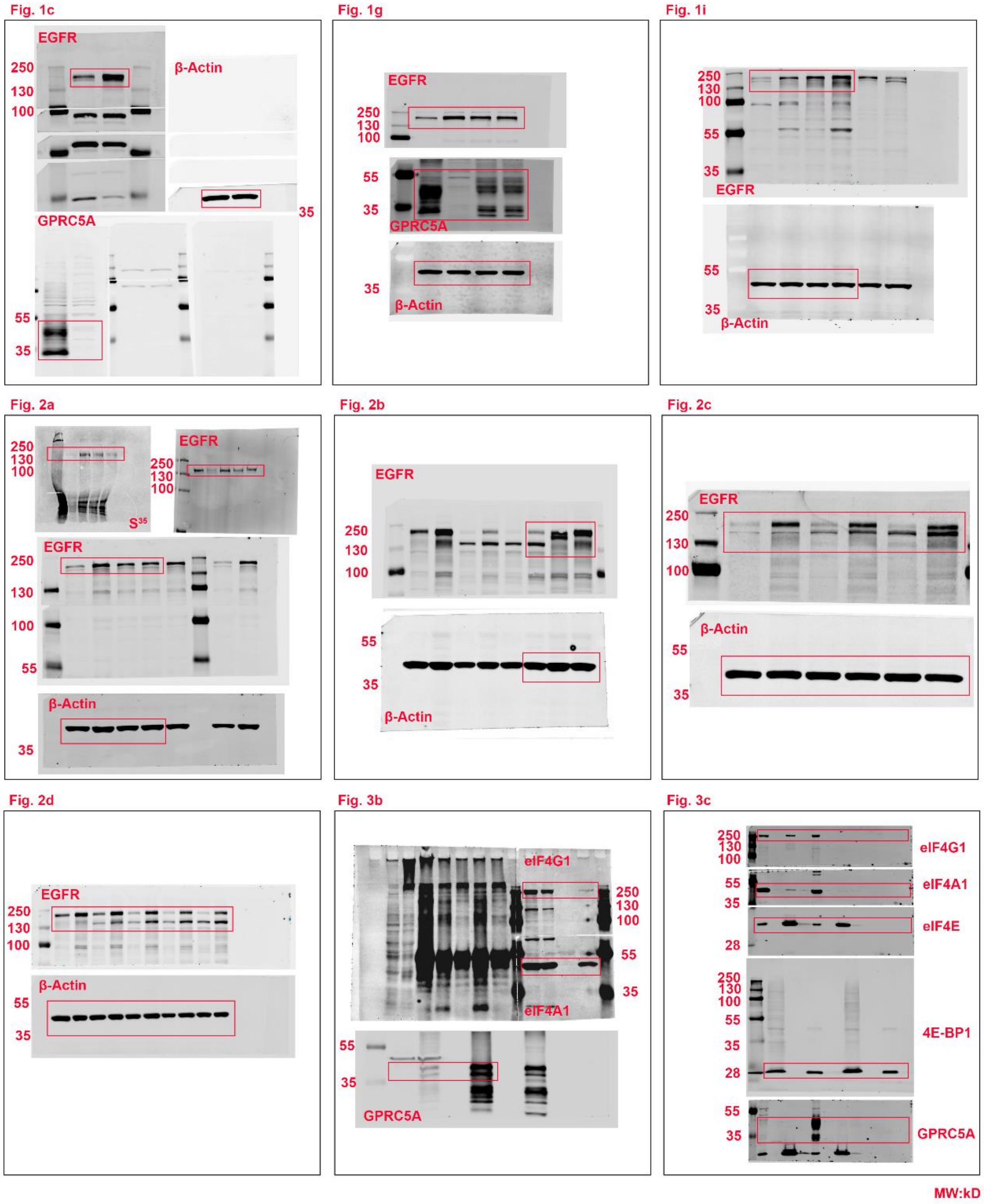


**Supplementary Fig. 5. Uncropped images of immunoblot.** Red boxes show cropped regions.

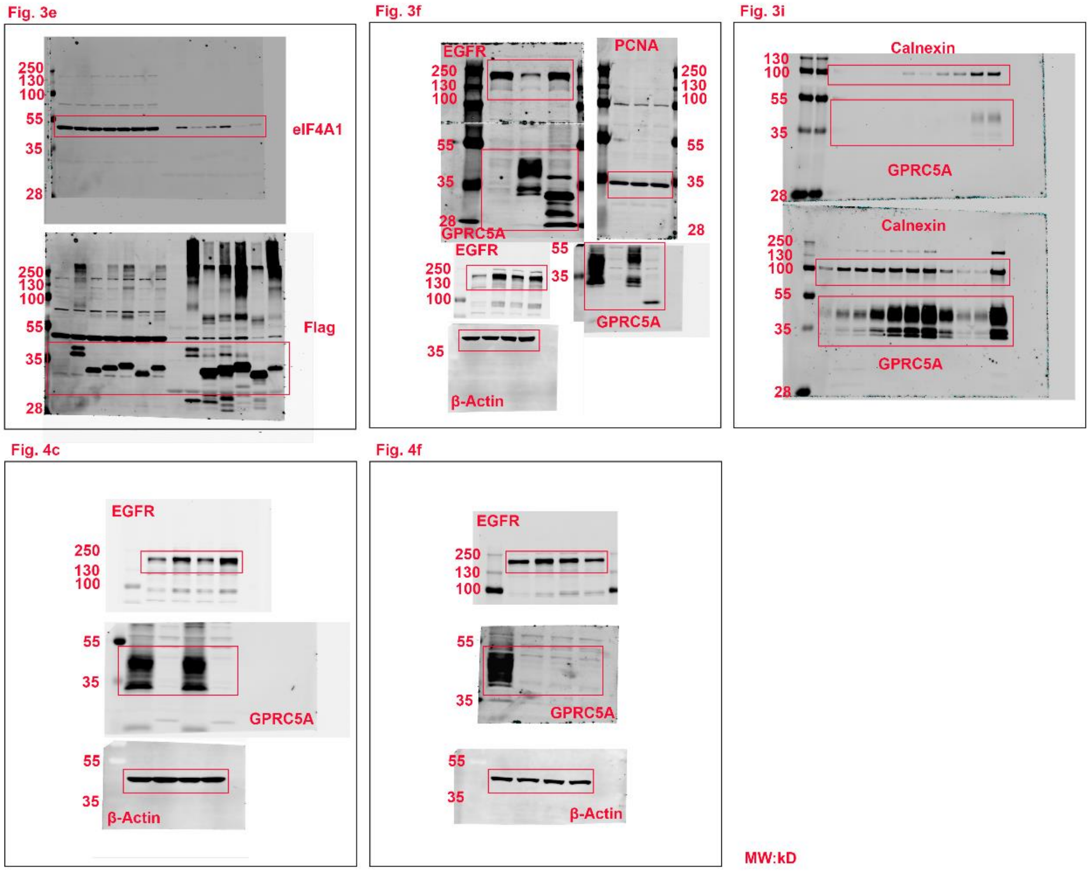


**Supplementary Fig. 5.** Continued.
